# Evolutionary constrained genes associated with autism spectrum disorder across 2,054 nonhuman primate genomes

**DOI:** 10.1186/s13229-024-00633-1

**Published:** 2025-01-23

**Authors:** Yukiko Kikuchi, Mohammed Uddin, Joris A. Veltman, Sara Wells, Christopher Morris, Marc Woodbury-Smith

**Affiliations:** 1 Biosciences Institute, Newcastle University, Newcastle upon Tyne, UK; 2 Center for Applied and Translational Genomics (CATG), Mohammed Bin Rashid University of Medicine and Health Sciences, Dubai, UAE; 3 GenomeArc Inc, Mississauga, ON Canada; 4 MRC Centre for Macaques, Salisbury, UK; 5 Mary Lyon Centre at MRC Harwell, Oxfordshire, UK; 6 Translational and Clinical Research Institute, Newcastle University, Newcastle upon Tyne, UK; 7 Department of Psychiatry, Queen’s University, Kingston, ON Canada

**Keywords:** Autism spectrum disorder, Primate model, Whole genome sequencing, Genetic constraint, GSEA

## Abstract

**Background:**

Significant progress has been made in elucidating the genetic underpinnings of Autism Spectrum Disorder (ASD). However, there are still significant gaps in our understanding of the link between genomics, neurobiology and clinical phenotype in scientific discovery. New models are therefore needed to address these gaps. Rhesus macaques (*Macaca mulatta*) have been extensively used for preclinical neurobiological research because of remarkable similarities to humans across biology and behaviour that cannot be captured by other experimental animals.

**Methods:**

We used the macaque Genotype and Phenotype (mGAP) resource consisting of 2,054 macaque genomes to examine patterns of evolutionary constraint in known human neurodevelopmental genes. Residual variation intolerance scores (RVIS) were calculated for all annotated autosomal genes (*N* = 18,168) and Gene Set Enrichment Analysis (GSEA) was used to examine patterns of constraint across ASD genes and related neurodevelopmental genes.

**Results:**

We demonstrated that patterns of constraint across autosomal genes are correlated in humans and macaques, and that ASD-associated genes exhibit significant constraint in macaques (*p* = 9.4 × 10^− 27^). Among macaques, many key ASD-implicated genes were observed to harbour predicted damaging mutations. A small number of key ASD-implicated genes that are highly intolerant to mutation in humans, however, showed no evidence of similar intolerance in macaques (*CACNA1D*, *MBD5*, *AUTS2* and *NRXN1*). Constraint was also observed across genes associated with intellectual disability (*p* = 1.1 × 10^− 46^), epilepsy (*p* = 2.1 × 10^− 33^) and schizophrenia (*p* = 4.2 × 10^− 45^), and for an overlapping neurodevelopmental gene set (*p* = 4.0 × 10^− 10^).

**Limitations:**

The lack of behavioural phenotypes among the macaques whose genotypes were studied means that we are unable to further investigate whether genetic variants have similar phenotypic consequences among nonhuman primates.

**Conclusion:**

The presence of pathological mutations in ASD genes among macaques, along with evidence of similar genetic constraints to those in humans, provides a strong rationale for further investigation of genotype-phenotype relationships in macaques. This highlights the importance of developing primate models of ASD to elucidate the neurobiological underpinnings and advance approaches for precision medicine and therapeutic interventions.

**Supplementary Information:**

The online version contains supplementary material available at 10.1186/s13229-024-00633-1.

## Background

Autism Spectrum Disorder (ASD) is a clinically defined, early-onset lifelong neurodevelopmental disorder that affects social, cognitive and communicative abilities. The health and well-being of autistic people is a priority among many governments worldwide. However, despite recent advances in ASD genetics, there are currently no effective biologically-derived treatment options that improve the health and wellbeing of autistic people. This underscores the importance of developing new animal models that can facilitate an understanding of both genetic and biological factors, which will inform and advance the efficacy and precision of potential therapeutic options to improve clinical outcomes.

Rhesus macaques (*Macaca mulatta*) have been extensively used for preclinical neurobiological research and macaques and humans share remarkable similarities in social and cognitive behaviours, and neurobiology that cannot be captured by other experimental animals [1–4]. More recently, primate species have been used to infer pathogenicity of mutations in humans, thereby facilitating better diagnostic accuracy in patients with genetic disorders [5]. Rodent models, although powerful, are insufficient as they lack primate-specific frontal brain structures that are critical for social and cognitive behaviours associated with ASD [2, 4, 6]. Consequently, a primate model could provide important information to clarify the evolutionary divergent mechanisms that occurred between rodents and humans. For instance, macaques have forward-looking eyes and their eye movement patterns are similar to those of typically developing children who attend preferentially to features of social or emotionally arousing stimuli [7–10]; such gaze behaviours are abnormal in ASD [11–14]. Furthermore, social behaviours among macaques can be quantitatively assessed and characterised in a way similar to ASD screening in humans, by using assessments such as the macaque Social Responsiveness Scale (SRS)-Revised (mSRS-R) [15, 16], which is derived from the human SRS [17, 18]. Consistent with the distribution of SRS scores in the general human population, the scores among macaques are also continuously distributed. Moreover animals with SRS scores greater than 1.5 SD above the mean exhibit a greater burden of autism-like traits [16].

Genetically altered nonhuman primate models of ASD have been generated to target single-genes that are implicated in ASD, such as *SHANK3* [19, 20] and *MECP2* [21, 22] in which abnormal social and repetitive behaviours, sleep disturbance and impairments in cognitive function are observed. Although single gene knockout models may work well for characterising the pathological mechanisms in individual genes, given the polygenic nature of ASD, genome-wide studies are crucial. It is advantageous that there is now a robust rhesus reference genome which provides a framework for evaluating mutational characteristics among macaque genes that are associated with human pathology. Recently a large macaque genomic database, the macaque Genotype and Phenotype (mGAP) resource, has become available, providing a catalogue of annotated macaque variant data derived from exome and whole genome sequencing [23].

In this study, we used the mGAP resource to examine genetic constraint among ASD-associated autosomal genes across 2,054 macaque genomes. We hypothesised that macaques would show similar genetic constraint to humans among orthologous genes, consistent with their likely importance in brain development, and consequently consistent with the likelihood of pathological consequences from those variants predicted to be damaging. As a corollary, we extended our analyses to examine constraint among other neurodevelopmental genes associated with epilepsy, intellectual disability (ID) and schizophrenia.

## Methods

### Rhesus macaque variant catalog (mGAP)

The Macaque Genotype and Phenotype Resource (mGAP) (https://mgap.ohsu.edu/) is an open access database of exome and whole genome sequencing from macaques housed at a number of primate research centres, but principally from a multi-generational outbred cohort of macaques bred at the Oregon National Primate Research Center. The iteration used in our analyses (v2.0) includes data from 2,054 Indian and Chinese-origin macaques, with additional limited phenotypes comprising basic metabolic parameters. The mGAP repository comprises animals genotyped by a variety of methods. At the time of our study, the dataset included 87% animals who underwent whole genome sequencing and 13% whole exome sequencing. Platform specific information is unavailable. Sequence data are analysed by the mGAP team using a modified version of the Broad Institute/GATK SNP and Indel Discovery Best Practices adapted for use with macaque data. All v2.0 data are aligned to the Mmul_10 reference genome [24]. The rhesus macaque reference genome is now of a very high standard, with few gaps and an average coverage of 99.8% [24]; more than 99% of genes are represented. The mGAP v2.0 release contains variants passing a quality filtering process including the use of Mendelian inheritance for validation.

### Clinical gene sets

To derive clinical gene sets for ASD and other NDDs (neurodevelopmental disorders i.e., epilepsy, intellectual disability [ID], and schizophrenia [SZ]), we used the DisGeNET database of curated genes and variants (https://www.disgenet.org/). These genes and variants are collated from different data sources, including those extracted by text mining the scientific literature [25]. Genes and variants are annotated in various ways, including the addition of metrics that demonstrate the strength of disease-gene/variant associations. DisGeNET is regularly updated (October 2021 update used for current analyses) and provides metrics that allow researchers to classify genes according to level of evidence. The final lists of DisGeNET genes comprised 793 ASD genes, 1,570 ID genes, 871 epilepsy genes and 2,018 schizophrenia genes. Separately, we used the latest version of the Simons Foundation Autism Research Initiative (SFARI) SFARI-Gene platform to download a list of ASD-associated genes [26]. This list comprised 729 genes. There were 292 genes occurring in both SFARI and DisGeNET ASD gene lists. Gene lists were accessed and downloaded on the 13th February 2023.

Gene lists such as those of SFARI and DisGeNET are curated from a variety of sources and comprise genes with different degrees of evidence for pathogenicity. The evidence for some genes is much stronger than others, and SFARI usefully groups genes into categories 1 (High Confidence), 2 (Strong Candidate), and 3 (Suggestive Evidence), according to level of evidence. DisGeNET similarly provides metrics that allow their curated lists to be ranked according to evidence. Given the exploratory nature of our study we decided to include all identified genes in the lists, but recognise that this may be over-inclusive. Consequently, we refer to the broader category of genes as ‘ASD-associated genes’, and only reserve the term ‘ASD-implicated genes’ for SFARI Category 1 genes.

### Quality screening of SNVs

We used bcftools, vcftools and R scripts for all analyses (all R scripts are available in our OSF repository, and related files are available as Supplementary Files). We first generated summary statistics for variant quality and variant depth and excluded all variants with a quality score of < 30, and all variants whose depth was < 10 or > 50. We did not exclude variants by their minor allele frequency (MAF) to avoid excluding any variants that might potentially be of significance. For the purpose of this current analysis, we focussed on only single nucleotide variants (SNVs), therefore excluding indels from all downstream analyses. We also focussed on only the autosomes.

### Genetic constraint in human and macaque genome

#### Residual variation intolerance score (RVIS) analysis

To investigate whether associated genes for ASD and other NDDs were subject to constraint against the accumulation of damaging mutations in the macaque genome, we generated gene-by-gene Residual Variation Intolerance Scores (RVIS) for macaque genomes, which uses an intolerance ranking system for each gene as described by Petrovski [27]. Briefly, this constraint metric is calculated by regressing the total number of functional variants (i.e., common missense and loss of function SNVs) in a gene on the total number of protein-coding variants in that gene. The studentised residuals (mean = 0) are then taken as the RVIS, which provides a measure that captures departure from the average mutational burden. When RVIS = 0, the gene has the average number of common variants given its total mutational burden; when RVIS < 0, the gene has fewer common variations than predicted (i.e., more constrained, mutation intolerant); when RVIS > 0, it has more common variation than predicted (i.e., less constrained, mutation tolerant). Among macaque data, two genes (*MUC16*, *LOC106997451*) with RVIS scores greater than 11 were removed from the analysis as potential outliers. Neither of these genes is deemed ‘essential’ according to Blomen et al.’s [28] and Godini at al.’s [29] ‘essentialome’, and neither feature in the SFARI or DisGeNET gene lists for the NDDs investigated (see below). Applying the same criterion to human RVIS data [27] resulted in 5 genes (*MUC16*, *MUC17*, *MUC5B*, *AHNAK2*, *FLG*) being excluded. None of these are known NDD genes.

We undertook analysis of group differences in RVIS score using t-tests. In order to examine the magnitude of constraint differences between groups, we also undertook logistic regression. All analyses were implemented in R, with the code available in our repository.

#### Gene set enrichment analysis (GSEA) analysis

We next undertook Gene Set Enrichment Analysis (GSEA) using the hypeR package in R [30]. hypeR implements the Kolmogorov-Smirnov test to determine whether a set of genes (in our case, our curated ASD and NDD genes) are randomly distributed across a ranked list (in our case, all autosomal genes), or instead, are preferentially clustered towards either end, as measured by calculation of an ‘enrichment score (ES)’. Through permutation of the ranked data, a null distribution of enrichment scores can be generated allowing a nominal p-value to be produced. As a corollary, we also undertook a gene set over-representation analysis using the same hypeR package. This method implements a hypergeometric test, which we used to examine the over-representation of ASD and other NDD gene sets among the top 2% constrained genes. These constrained genes were identified by first ranking genes and then identifying the top 363 genes (18,166 * 0.02).

### Analysis of predicted-damaging mutations

Where possible, variants in mGAP have been lifted to the human genome (hg38) and annotated using a variety of bioinformatic tools, as described on the mGAP website. In this way, a list of predicted damaging variants has been generated by the mGAP team, comprising those variants that either (i) are identical to a ClinVar allele annotated as pathogenic, (ii) are predicated damaging according to Polyphen2 score, or (iii) predicted as high impact by SnpEff. Using the mGAP ‘predicted_damaging’ file for v2.0, we examined both (i) the number of ASD-associated genes (SFARI and DisGeNET curated) that harboured one or more predicted-damaging mutations in macaques and (ii) whether any of these predicted damaging mutations were identical to ASD mutations listed in DisGeNET and SFARI. The UCSC’s LiftOver tool was used to change coordinates to and from the macaque MMul_10 and hg38 builds. Scripts used for these analyses are all available.

## Results

We undertook analyses on mGAP release 2.0, made available on the 18th January 2021. All samples are aligned to the Mmul_10 reference genome, and variants called using GATK’s GenomicsDB pipeline as detailed on the mGAP website. This release included 2,054 macaques, among whom basic phenotype data are available on 1,299 indicating the majority to be of Indian origin (*N* = 1,206) with the remainder Chinese (*N* = 13), mixed Indian/Chinese (*N* = 8) or unknown origin (*N* = 71). There were 703 male and 596 female macaques among those with available sex data. We first filtered variants according to variant quality and depth as described in the Methods. After filtering, a total of 37,503,663 variants remained across the 20 macaque autosomes comprising 32,507,418 SNPs (86.7%) and 4,996,245 Indels (13.3%). The total number of variants annotated missense according to SnpEff is 370,683. Additional File 1 Supplementary Table 1 shows metrics before and after our filtering steps. We excluded Indels and undertook analysis only on SNPs.

Genetic constraint was calculated using RVIS across all 18,168 genes as described in Methods. Using human RVIS data from Petrovski [27], we compared the RVIS in 18,166 genes in the human and macaque genome and identified a significant correlation between human and macaque RVIS scores (Additional File 1 Supplementary Fig. 1, Pearson’s product-moment correlation = 0.39 [95% CI = 0.38–0.41], *P* < 2.2e^− 16^). To ensure the validity of RVIS as a measure of constraint, we also investigated whether the RVIS scores in macaques correctly identify those genes that are deemed ‘essential’ in humans, as identified by Blomen et al. (1,734 genes) [28] and Godini et al. [29] (1,586 genes) (Additional File 2 Table 1: Blomen-2015-core-essentialome; Additional File 2 Table 2: Godini − 2015-essential-genes). Using logistic regression, we identified that in both instances the essential genes were significantly more constrained than non-essential genes (‘Blomen genes’: Beta = -0.17, SE = 0.04, *P* = 1.38e^− 05^; ‘Godini genes’: Beta = -0.26, SE = 0.04, *P* = 4.19e^− 11^).

**Fig. 1:**
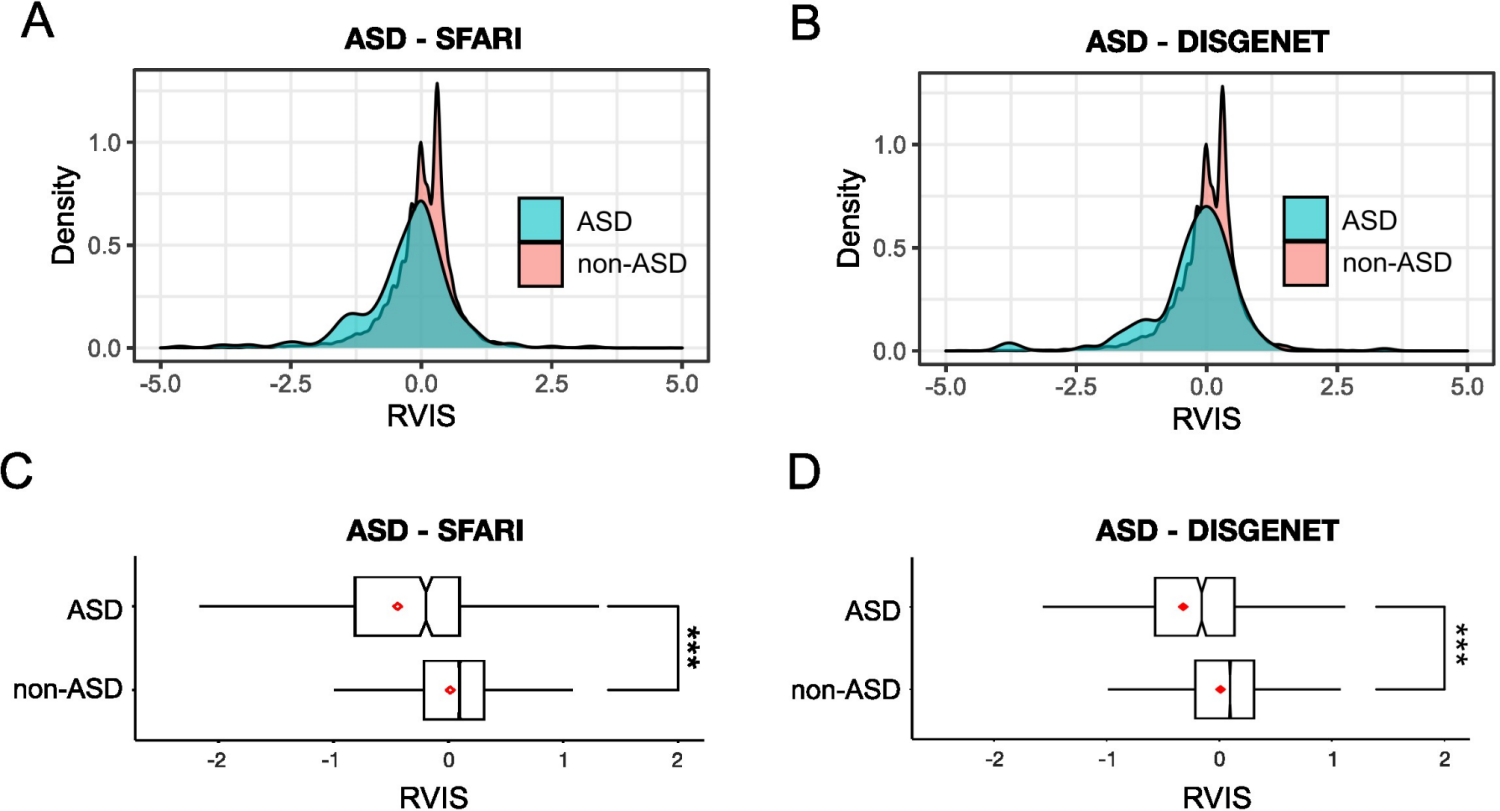
**A**. Density histogram of the Residual Variation Intolerance Scores (RVIS) for SFARI ASD genes (blue, *N* = 729) and non-ASD (red, *N* = 17,437) genes. The RVIS scores are normalised and hence centre on a mean of 0. Delimiters on x-axis range from − 5.0 to 5.0. **B**. The descriptions are the same as **A** for the DISGENET ASD genes (blue, *N* = 793) and non-ASD (red, *N* = 17,373) genes. **C**. Box and whisker plots depicting the distribution of RVIS scores with median (red line) for SFARI ASD and non-ASD genes. Delimiters on x-axis range from − 1.5 to 1.5. *** *P* < 0.001 (t test). **D**. Same as C using the DisGenet ASD genes

### Genetic constraint of ASD-associated genes

We examined the distribution of RVIS scores among SFARI (Fig. 1A and C) and DisGeNET (Fig. 1B and D) ASD-associated genes respectively. In both instances, the RVIS scores were indicative of greater constraint among ASD-associated genes compared to the background genome (SFARI: *N* = 729, mean = -0.44, SD = 1.10; non-ASD: *N* = 17,437, mean = 0.02, SD = 0.67, *P* = 9.4 × 10^− 27^, OR = 0.53; DisGenet: *N* = 793, mean = -0.32, SD = 0.88 ; non-ASD: *N* = 17,373, mean = 0.01, SD = 0.67, *P* = 4.6 × 10^− 24^, OR = 0.61). (Additional File 1 Supplementary Table 2).

**Fig. 2:**
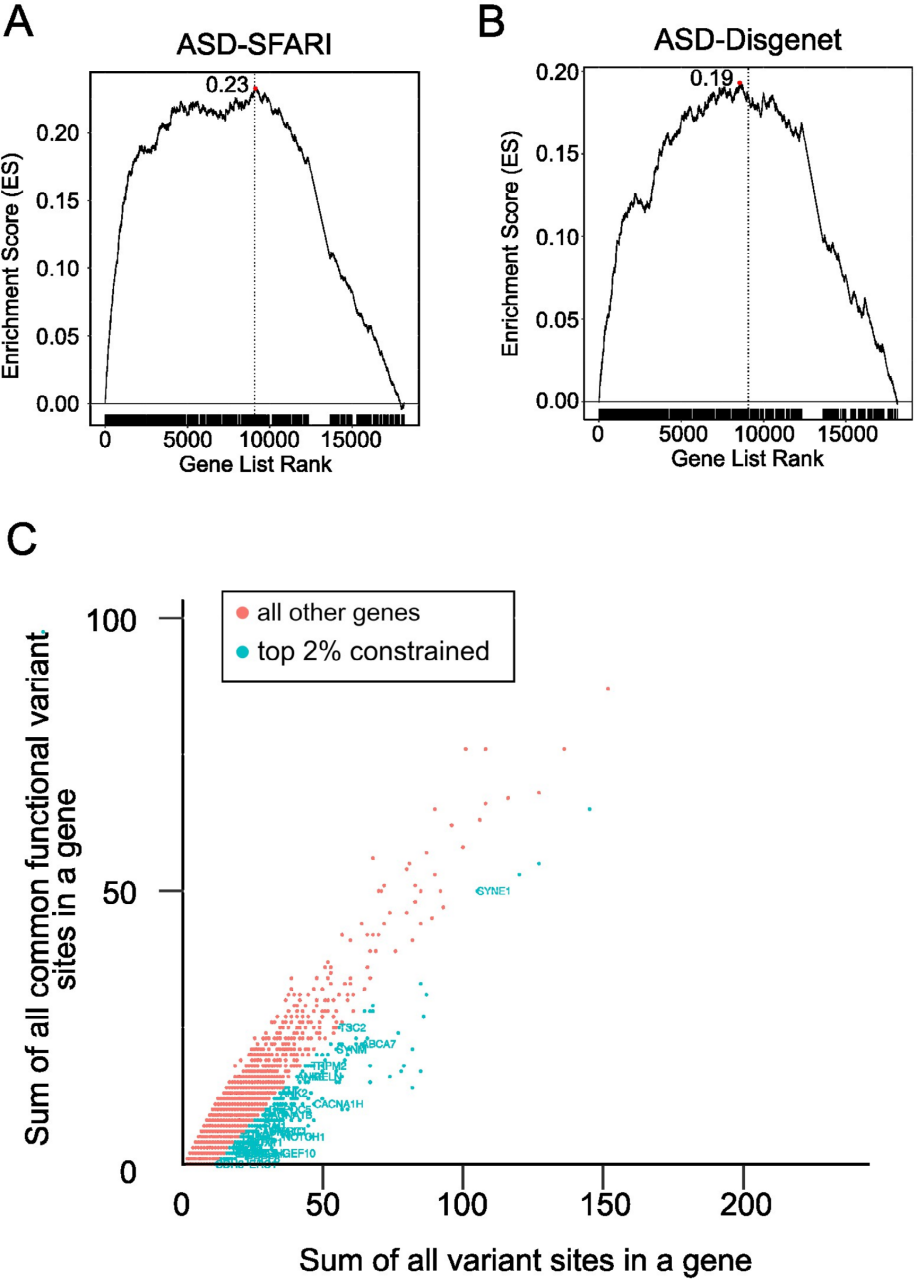
Gene Set Enrichment Analysis (GSEA) using ASD SFARI gene set (**A**) and DisGeNET ASD gene set (**B**). The x-axis denotes all genes ranked by the RVIS. The black vertical line indicates the ASD set genes. The y-axis is the Enrichment Score (ES) which represents the degree (i.e., the maximum deviation from zero) to which a set of genes is over-represented at the top of the ranked list accordingly to the RVIS. **C.** The scatter plot of RVIS scores for ASD-associated genes. The blue plots are for the 2% extreme of the distribution i.e., most intolerant in which the annotated plot indicates the DisGeNET ASD set genes. The red plots indicate 98% of the rest of the genes

Next, Gene Set Enrichment Analysis (GSEA) was performed to investigate whether ASD-associated genes were enriched among the most constrained genes ranked according to their RVIS. As expected, ASD-associated genes showed evidence of enrichment among the more constrained genes (ES for ASD-SFARI = 0.23; ES for ASD-DisGenet = 0.19, Fig. 2A-B). As a corollary, we also investigated whether ASD genes were over-represented among the top 2% constrained genes (i.e., those genes with most negative RVIS, *N* = 369 genes) (Fig. 2C). In each case, significant over-representation was observed in the ASD-associated genes (ASD-DisGeNet, *P* < 9.6 × 10^− 13^, ASD-SFARI, *P* < 2.8 × 10^− 24^, Additional File 1 Supplementary Table 3).

Next, we investigated whether any of the predicted damaging mutations identified by mGAP’s annotation pipeline (*N* = 128,705 variants) overlapped with known ASD variants as curated by DisGeNET (*N* = 332 variants). The result shows that none of the macaque predicted damaging variants overlapped the known ASD variants. However, the majority of ASD genes included at least one predicted-damaging variant (Table 1).

**Table 1:** Counts of predicted damaging variants that overlap variants associated with each studied NDD

	Number of genes in DisGeNeT database	Number with one or more mGAP predicted damaging variants	Proportion of disease gene with one or more predicted damaging variant
ASD	1,071	823	76.8%
ID	2,165	1,732	80.0%
Epilepsy	1,215	933	76.8%
Schizophrenia	2,872	2,098	73.1%

### Genetic constraint among other neurodevelopmental disorders

**Fig. 3:**
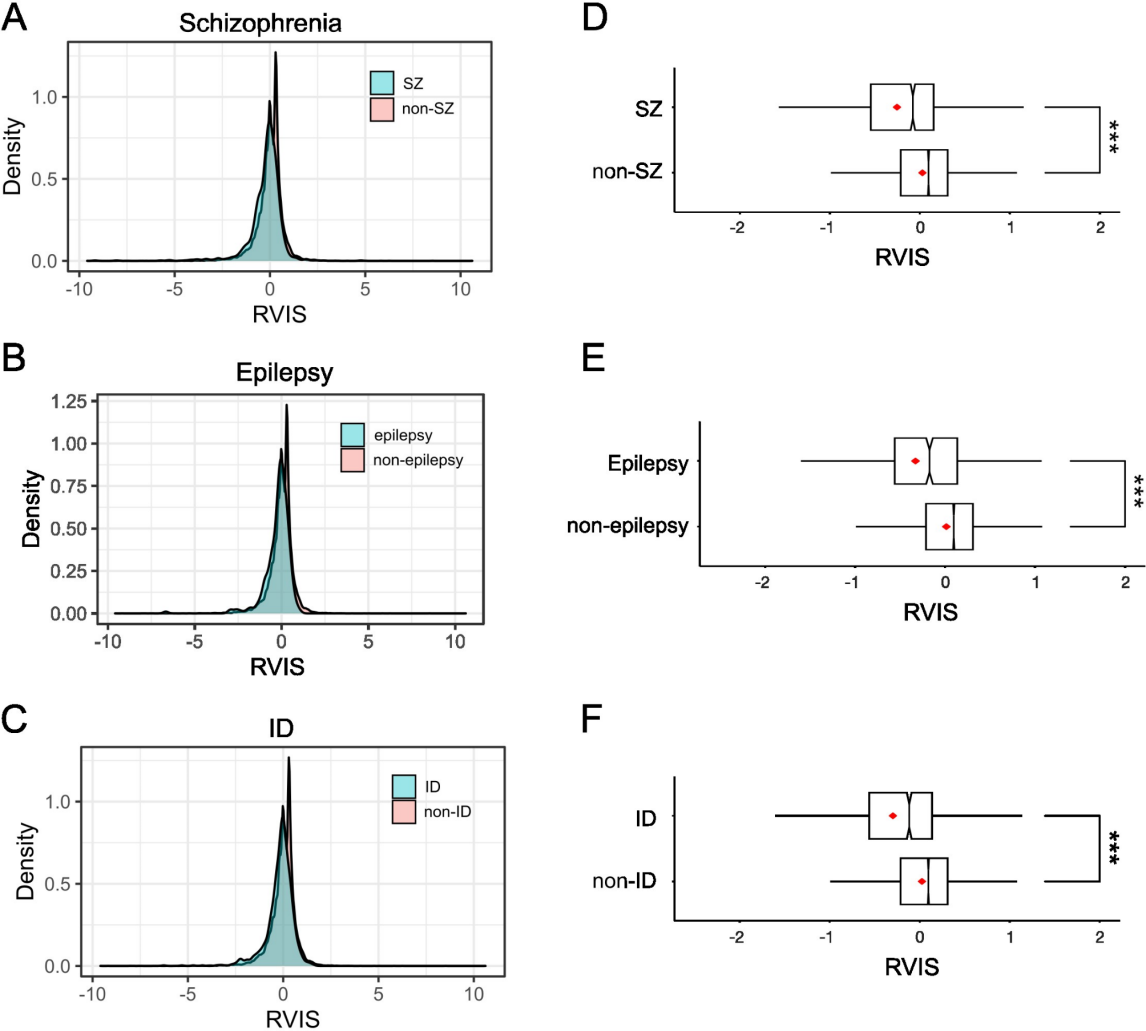
**A**. Density histogram of the Residual Variation Intolerance Scores (RVIS) for DisGeNET schizophrenia (**A**), epilepsy (**B**) and intellectual disability (ID) genes (**C**) (coloured in blue) and non-disorder associated genes (red). The RVIS scores are normalised and hence centre on a mean of 0. Delimiters on x-axis range from − 5.0 to 5.0. **B**. The descriptions are the same as **A**. **D-F**. Box and whisker plots depicting the distribution of RVIS scores with median (red line) for schizophrenia (**D**), epilepsy (**E**) and ID (**F**) DisGeNET genes. Delimiters on x-axis range from − 1.5 to 1.5. *** *P* < 0.001 (*t*-test)

Next, we examined constraint scores for other NDD gene sets to test whether RVISs for different NDD gene sets were consistent with the evidence of greater constraint. The RVIS scores were as follows: schizophrenia (mean: -0.25, SD: 0.85), ID (mean: -0.30, SD: 0.84) and epilepsy (mean: -0.33, SD: 0.79). These differences were significant for all disorders studied (Additional File 1 Supplementary Table 2). The RVISs were indicative of greater constraint across all gene sets (ID: *p* = 1.1 × 10^− 46^, OR = 0.58; epilepsy: *p* = 2.1 × 10^− 33^ OR = 0.60; schizophrenia: *p* = 4.2 × 10^− 45^, OR = 0.61, Fig. 3).

**Fig. 4:**
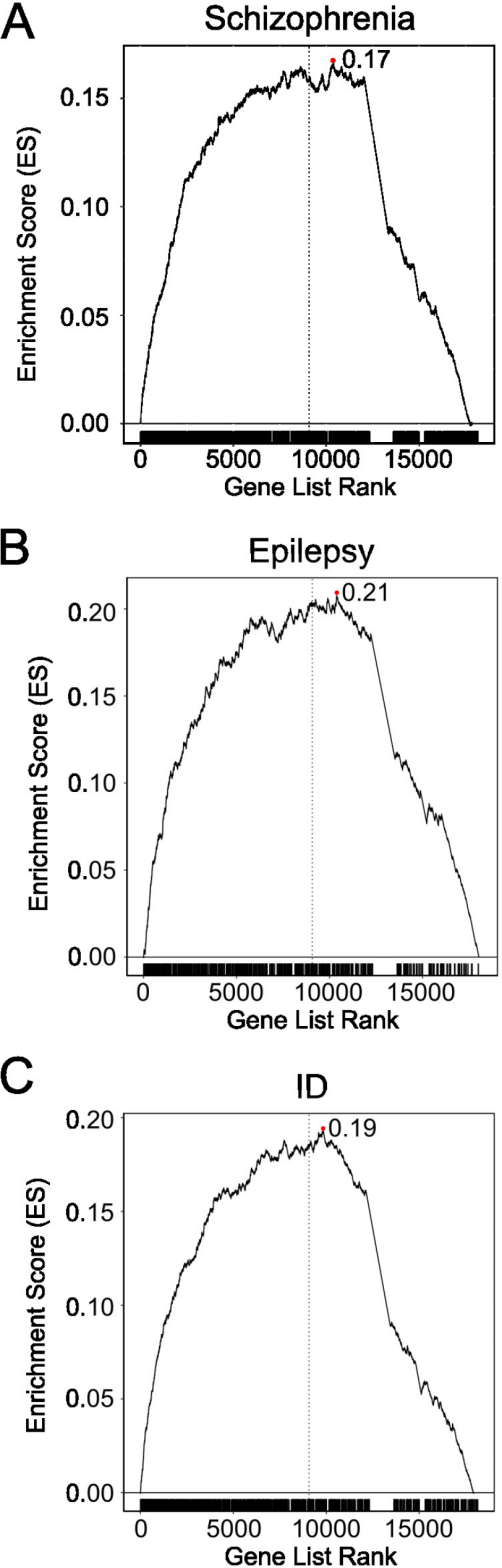
**A**. GSEA results using the set genes for schizophrenia (**A**), epilepsy (**B**) and ID (**C**). The descriptions are the same as Fig. 2A

We also undertook GSEA on these neurodevelopmental disorders. Consistent with our prediction, all gene sets showed evidence of enrichment towards the more constrained ‘end’ of the ranked genes (ES: 0.17 for schizophrenia; 0.2 for epilepsy; 0.19 for ID, Fig. 4, Supplementary Table 4 for each diagnosis). As a corollary, we also investigated whether NDD genes for each diagnosis were over-represented among the top 2% constrained genes. In each case, significant over-representation was observed (ID: *P* < 1.7 × 10^− 19^, Epilepsy: *P* < 8.9 × 10^− 12^, Schizophrenia: *P* < 5.9 × 10^− 16^; Additional File 1 Supplementary Table 3).

To investigate whether any of the predicted damaging mutations identified by mGAP’s annotation pipeline (*N* = 128,705) overlapped with known NDD variants, as curated by DisGeNET, we examined the frequency of the predicted damaging mutations. After lifting over co-ordinates, two predicted damaging variants are known schizophrenia variants in humans (rs2241621: 81270732 [hg38], *STON2*, A > C, T, missense variant; rs16969968: 78590583 [hg38], *CHRNA5*, G > A, missense variant), one is a known ID variant (rs587777623: 686986 [hg38], *DEAF1*, G > A, missense variant) and one a known epilepsy variant (rs28941773: 120739168 [hg38], *ACADS*, C > T, missense variant). As was the case for ASD genes (see above), the majority of genes across these disorders harboured one or more predicted-damaging mutations (ID: 80.0%, epilepsy: 76.8%, schizophrenia: 73.1%, Table 1).

### Genetic constraint among key NDD genes

Given the inclusive nature of these NDD gene sets, i.e., the relatively low threshold for any particular gene to be identified as disorder-associated, we further refined our gene list to comprise only those genes that overlapped among all four disorders (*N* = 101 genes, see Additional File 2 Table 3 – Overlapping NDD genes). The importance of the genes in this list is demonstrated by their key role in certain brain processes (Additional File 2 Table 4 - g: profiler mmulatta_intersections.csv).

**Fig. 5:**
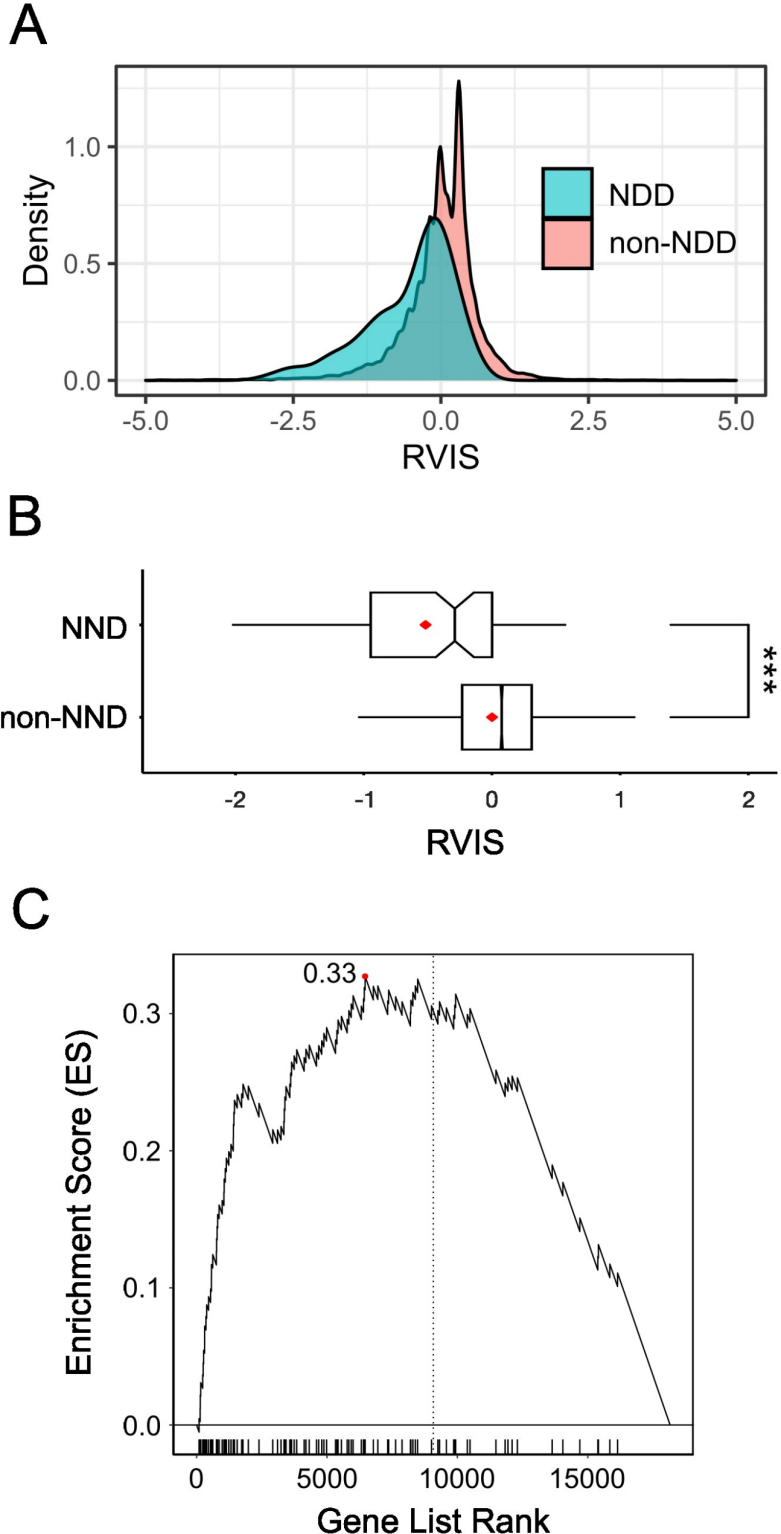
**A**. Density histogram of the Residual Variation Intolerance Scores (RVIS) for NDD (*N* = 101, blue) and non-NDD (*N* = 18,065, red) genes. The RVIS scores are normalised and hence the centre on a mean of 0. **B**. The box plots for any NDD genes (i.e., ASD, epilepsy, ID, schizophrenia) and non-NDD genes. The descriptions are the same as Fig. 2C and D. Box plots **C**. GSEA for the overlapped NDD genes

Figure 5 shows the distribution of RVIS scores in NDD-associated genes and non-NDD genes (NDD: *N* = 101, mean = -0.52, SD: 0.75; non-NDD: *N* = 18,065, mean = 0.00, SD: 0.68, *P* = 4.0 × 10^− 10^, OR = 0.59). (Additional File 1 Supplementary Table 2).

We examined the burden of predicted damaging mutations in these genes in the macaque data. In total, 91 of the 101 genes (90.1%) had one or more predicted damaging variants, with a total of 809 variants across all genes (Additional File 2 Table 5 – Overlap Hits). We conclude, therefore, that despite the constraint against mutation among these NDD-associated genes, macaques still harbour predicted damaging mutations, with potential phenotypic consequences. By way of illustration, Table 2 shows the 20 predicted damaging variants with the largest Combined Annotation Dependent Depletion (CADD) scores [31]. These scores are based on the integration of multiple annotation metrics into one score which earmarks likely pathogenicity. A score of 30 or more are deemed likely deleterious, indexing the most 0.1% pathogenic substitutions across the genome. Similarly, a score of 20 or more indexes the top 1% deleterious substitutions. Given these high CADD scores, and the fact that these genes are largely well-recognised as key NDD genes, it might be predicted that in humans these mutations would have clinical neurodevelopmental consequences.

**Table 2:** Predicted damaging variants in NDD genes and their CADD scores

Gene	Chr	Start (hg19)	End (hg19)	CADD
*RAI1*	chr16	17625726	17625726	45
*CNTN2*	chr1	62585295	62585295	40
*MAPK1*	chr10	31173015	31173015	37
*CACNA1D*	chr2	109940657	109940657	37
*SLC52A2*	chr8	144641018	144641018	37
*LRP2*	chr12	56308216	56308216	36
*PIK3CG*	chr3	133216881	133216881	36
*CNTN2*	chr1	62590334	62590334	35
*ERBB4*	chr12	98730326	98730326	35
*GRIK4*	chr14	113885581	113885581	35
*RELN*	chr3	129923111	129923111	35
*TRIO*	chr6	14733837	14733837	35
*NTRK1*	chr1	94089606	94089606	34
*MTOR*	chr1	213265857	213265857	34
*RAI1*	chr16	17644443	17644443	34
*RELN*	chr3	129793522	129793522	34
*CD38*	chr5	14788455	14788455	34
*TRIO*	chr6	14752487	14752487	34

### Cross-species comparison of highly constrained genes in humans and macaques

A number of genes associated with human NDDs were characterised by high constraint scores in both humans and macaques, as evidenced by their RVIS scores more than two standard deviations from the mean (*N* = 5 genes with macaque: RVIS < -1.47, human: RVIS < -1.97) [Additional File 2 Table 6: “RVIS scores human and macaque overlapping NDD genes”]. These include *TRIO*,* RAI1*,* MTOR*,* TSC2* and *RELN*. All these genes are implicated in NDDs, two of which are implicated in specific syndromes, namely *RAI1* (Smith-Magenis Syndrome) and *TSC2* (Tuberous Sclerosis) Both Smith-Magenis syndrome and Tuberous Sclerosis are known to be associated with moderate to severe intellectual disability in humans and are also characterised by other medical and neuropsychiatric features. These genes and their mGAP annotated predicted pathogenic mutations are illustrated in Additional File 2 Table 7 “NDD_variants_top5_rvis_human_macaque”. Interestingly, a further four NDD genes were characterised by high constraint scores in humans but not in macaques. These include *CACNA1D*, *CNTNAP2*, *MBD5*, *AUTS2* and *NRXN1* (Additional File 2 Table 8 - NDD_variants_top5_rvis_human_notmacaque).

## Discussion

In this study, we investigated the mutational burden in ASD and other neurodevelopmental genes among macaques in two fundamental ways. First, we determined whether genes associated with ASD and a number of related neurodevelopmental disorders in humans show evidence of evolutionary constraint against the accumulation of mutations. Second, we examined whether any of those macaque mutations that are predicted damaging in humans overlapped with known ASD and other NDD-associated mutations in humans. Our analysis identified evidence of evolutionary constraint against the accumulation of mutations across all ASD and other NDD gene sets, indicating that, in macaques, as in humans, these genes have important functions. We also demonstrated that predicted damaging mutations were still quite common in all NDD genes, including those that overlap across all NDD disorders and are presumably the most important. Again, given their predicted damaging nature, it might be anticipated that these variants would have phenotypic consequences. Warren et al. [24] examined sequence diversity in a sample of 853 macaques and identified thousands of missense variants across the genome impacting genes associated with human disease. Our results are largely consistent with their study, which analysed sequence diversity in this same dataset. Unlike our study, which set out to examine constraint across neurodevelopmental genes in different ways, theirs specifically examined the frequency of missense variants in these genes compared to all genes and found them to be depleted for missense variants. Nevertheless, our investigation did identify the fact that these NDD genes do harbour missense and other potentially damaging variants that could have phenotypic consequences, as discussed subsequently.

We also demonstrated that, despite constraint, macaques harbour predicted damaging mutations in key NDD genes. These predictions are based on mGAP’s own annotation derived from multiple sources of information after variants are lifted to the human genome, including established prediction tools (such as CADD and SIFT) and ClinVar annotations. These were supplemented with SnpEff annotation for predicted impact on protein-coding genes. Given this, in humans these variants would be expected to have phenotype consequences, although both variable expressivity and variable penetrance will confound the prediction of phenotypic consequences. Recent macaque studies have also shown that macaques with single gene editing or naturally-occurring mutations present a combination of phenotypic behaviours including social and learning impairment as well as repetitive behaviours [19, 21, 32, 33].

One further insight relates to the observation that a number of genes with strong evidence of constraint in humans were not similarly constrained in macaques. Some of these genes, such as *CACNA1D*, *MBD5*, *AUTS2* and *NRXN1* are key neurodevelopmental genes with high penetrance for clinical phenotypes. For example, copy number variation in *NRXN1*, which encodes a synaptic scaffolding protein, has a penetrance of 6.4% for schizophrenia and 26% for ASD or ID [34]. *MBD5*, a transcriptional regulator is implicated in ID, ASD, epilepsy and specific cognitive impairments [35]. CACNA1D, which encodes one of the L-type calcium channels is similarly associated with (SFARI Category 2) ASD, ID and epilepsy. Crucially, these genes encode important aspects of brain structure and function, so their constraint against mutations in humans, and the phenotype consequence contingent on mutations occurring, is unsurprising. It is curious, therefore, that similar constraint is not seen in macaques. Gao et al. (2023) also identified a small number of variants with strong evidence of pathogenicity in humans that appeared to be well-tolerated in NHPs, proposing that interactions within the genomic neighbourhood may be relevant. Another possibility is that constraint is more fine-grained than at the gene level. For example, previous research has examined patterns of constraint among Pfam protein domains, showing tolerance to be consistent across specific domains in different genes, and that evolutionary conservation is correlated between entropy measured from Pfam and ExAC [36]. Moreover, among some ID-associated genes, pathologic *de novo* mutations have been shown to cluster in specific protein domains [37]. Examining patterns of constraint in different ways among primate datasets may therefore offer further insight into pathophysiology in human NDDs.

Our decision to use the RVIS was based on our belief that it is a well-established measure of constraint, used across species, that provides an easily interpretable metric for readers. Another widely used metric is the pLI, which uses expectation-maximization to estimate intolerance to loss of function (LoF) variation specifically. Given the exploratory nature of this study, we were interested in casting the net wide beyond LoF to also include missense mutations. We also aimed to use a metric that would be straightforward to interpret and could inform future NHP studies investigating phenotypes in constrained NDD genes, as well as genes that demonstrate constraint in humans but not NHPs. Notably, discrepancies can arise between different methods used for measuring constraint. For example, we identified *CNTNAP2* as highly constrained, while gnomAD assigns a pLI of 0 to this gene, suggesting its tolerance to mutation. This anomaly can be attributed to several factors, primarily the specific population studied, the availability and interpretation of phenotypic data, and the relative lack of functional data [38]. It is also expected that metrics will be revised over time as more data accumulate. In clinical situations, it is essential that these metrics are not interpreted in isolation; however, in the research, they provide valuable insights into which genes should be prioritised. This is especially important in NHP research, which can be very costly to undertake.

Although our correlational analysis of human and macaque genes was significant, the value of 0.39 is lower than expected. Supplementary Fig. 1 illustrates the most of genes are clustered around the mean, and it may be the more peripheral genes that are driving this lower correlation. Notably, there are a small number of genes whose scores do not align in humans and macaques and these may be worthy of further investigation (examples include *DNAH10*, *TTN*). Additionally, given that the accumulation of mutations and its converse, are dynamically established through evolution, these differences may be indicative of the true impact of evolution on constraint across species, and may potentially provide insights into species-specific phenotypes.

Key differences at a transcriptomic [39] and cellular [40] level have also been observed across human and NHP species consistent with shared and unique dynamic developmental processes. In particular, the retention of duplicated genes is widespread across species, and likely plays a role in determining evolutionary pathways. The macaque reference genome has good coverage for segmental duplications (SDs) [24] that correspond to recent expansion in the major histocompatibility complex (MHC) and other gene families in this species. Although the frequency of SDs approximates humans, in macaques these SDs are more clustered. It is possible that the duplication of genes may result in copies with variable constraint.

Human studies have identified many genes associated with these NDDs; however, much is still not known about the neurophysiological, cognitive and other intermediate phenotypes. There are several limitations to human studies that may be overcome by shifting the focus to NHP-based research. One challenge has been the involvement of less able individuals with ID, ASD or epilepsy, who may be unable to follow instructions or comply with neuroimaging and electrophysiology, which are important tools for studying NDDs. It is important that such individuals are included in research, as they often represent the ‘purest’ forms of the disorder among whom there is wide consensus regarding diagnosis. However, recruiting such individuals and conducting research in an ethical manner has also been difficult, given the restrictions inherent in any research protocol.

New models are therefore needed. Up to now, NDDs have largely been studied in rodent models. However, rodents and humans have diverged significantly more than 70 million years ago, there are major differences in brain structure and function between the two species. This makes it difficult to recapitulate the research paradigms that capture clinical phenotypes for NDDs in rodents. NHP models offer many advantages over rodent models for studying NDDs. Macaques are a particularly promising model for studying NDDs as they share many similarities with humans in terms of social behaviours and have a well-characterised genome. Given that macaques are among our most recent NHP ancestors, we might anticipate that genes that are important in humans would also be important in macaques. It is therefore perhaps unsurprising that NDD genes show evolutionary constraint similar to humans. These genes clearly play a key role in core brain functions, as evidenced in our network analysis, and as such are presumably central to developmental brain processes. Moreover, given that the NDDs studied are all characterised by language abnormalities (including language pragmatics*)* and that humans have more advanced vocal communications than macaques, there may be reasons to predict that these genes might be associated with more fundamental vocal communication or language systems with a shared a common evolutionary origin. Indeed, recent studies have revealed evolutionarily conserved abilities [41, 42] and brain signatures [43–45] associated with language precursor in both humans and macaques. Given our findings, the role of these genes in brain development is more likely to be pre-linguistic and hence at a far more fundamental level than the traits that characterise these disorders. Consequently, these genes may play a less important role in the human specific ‘high-level’ manifestations of these disorders, which include aspects of social skills and language itself. What ultimately determines the clinical phenotype itself is downstream of the genes through their interaction with molecular neurobiology, neural circuits and connectivity through environment and other, as yet undetermined, factors.

Furthermore, the recent availability of a reference genome, a spatial transcriptomic atlas of the macaque cortex [46] enables cross-species comparison of humans and macaques in specific brain regions. However, a hypothesis is still needed to guide the selection of regions of interest associated with phenotypic behaviours. The recent comparative macaque and human studies have identified evolutionarily conserved cognitive systems within the prefrontal cortex [43–45], with the cognitive functions hypothesised to be impaired in ASD [47–49]. Additionally, human and macaque transcriptomic studies, including recent advances in single-cell genomics and spatial transcriptomic technologies, have revealed enriched gene expression of ASD-associated genes in the prefrontal cortex [50].

Furthermore, the usefulness of macaque Social Responsiveness Scales (mSRS) [15, 16], which is a screening questionnaire that can identify animals who demonstrate vulnerabilities indicative of ASD based on the SRS that is widely used clinically as a screening tool [51], has been illustrated by several studies, for example, significant cerebrospinal fluid (CSF) arginine vasopressin (AVP) was reported in low-social macaques and also in individuals with ASD [52]. Associations between social behaviours and whole genome sequence data in macaques have also been demonstrated [33].

This highlights the importance of developing a macaque model of ASD and the challenge now is to develop research paradigms that capture the characteristics of neurodevelopmental disorders in both clinical terms and in relation to the neurobiology of cognition that are immediately downstream of these clinical manifestations. Indeed, there is a burgeoning research examining social and communicative skills and aspects of behaviour and temperament among NHPs that will have implications for interpretation of data. For example, one study using heritability estimates has identified a preferential paternal transmission of social skills among male offspring [53], and another has established the heritable nature of anxious or inhibited temperament in multigenerational macaque families [54]. These studies demonstrate the ability to employ similar strategies to those that have been employed with human data, and, crucially, using phenotype measures and phenomenological discussions that overlap very closely with humans. ‘Translating’ the human characteristics of disorders such as ASD into animal behaviour is not straightforward, however, and it is hoped that further work in this area will now thrive in anticipation of the major contribution of NHPs in biomedical science.

### Limitations

The lack of behavioural and other phenotypes in the macaques studied limits the conclusions that can be drawn regarding the possible consequences of the pathological mutations described. Furthermore, other measures of evolutionary constraint are available, although research supports consistency between these different metrics. There are also limitations with gene lists used for particular disorders, given that these lists change over time with the accumulation of new evidence. It might be argued that these lists are overly inclusive, especially as we did not set an arbitrary threshold for inclusion but instead chose to include all associated genes in these lists. Given the exploratory nature of our study, this was a deliberate decision. To address potential over-inclusivity, we conducted analyses on genes overlapping between disorders (NDD genes), a list of about 100 genes among which are many of the most robust ASD-implicated and NDD genes.

## Conclusions

The presence of pathological mutations in NDD genes among macaques, and the evidence of similar constraint in these genes to humans, provides a strong rationale for further modelling genotype-phenotype relationships in macaques. Given their ability to model social cognition and other higher cognitive functions, and their more recent evolutionary separation from humans, macaques are an ideal model for translational purposes to further our understanding of the neurobiological underpinnings of ASD and other NDDs. Macaques also offer the opportunity to explore the nature of this relationship through mediating neuropsychological and neurofunctional modelling. This highlights the importance of this species as a prominent animal model to understand the neurobiological underpinning of ASD.

## Electronic Supplementary Material

Below is the link to the electronic supplementary material.

Supplementary Material 1

Supplementary Material 2

## Data Availability

The data used to generate the results and figures underlying the findings are publicly available on the Open Science Framework at https://osf.io/twg6v/. The data will be available in perpetuity in the event the corresponding authors leave Newcastle University Medical School.

## Abbreviations

ASD: Autism spectrum disorder
NDD: Neurodevelopmental disorder
ID: intellectual disability
RVIS: Residual Variation Intolerance Scores
GSEA: Gene Set Enrichment Analysis
NHP: Nonhuman primate
mGAP: macaque Genotype and Phenotype
SRS: Social Responsiveness Scale
