# Supplementary material for: Evolutionary constrained genes associated with autism spectrum disorder across 2,054 nonhuman primate genomes
Source: Mol Autism. 2025 Jan 23;16:5. doi: 10.1186/s13229-024-00633-1 (PMC11755938; doi:10.1186/s13229-024-00633-1)
Supplement: Supplementary file 1 — Supplementary Material 1 [file 13229_2024_633_MOESM1_ESM.pdf]

Additional File 1 for Kikuchi et al. *Evolutionary constrained genes implicated in autism spectrum disorder across 2,054 nonhuman primate genomes*

Supplementary Figure

Supplementary Figure 1: Correlation of human and macaque RVIS scores.

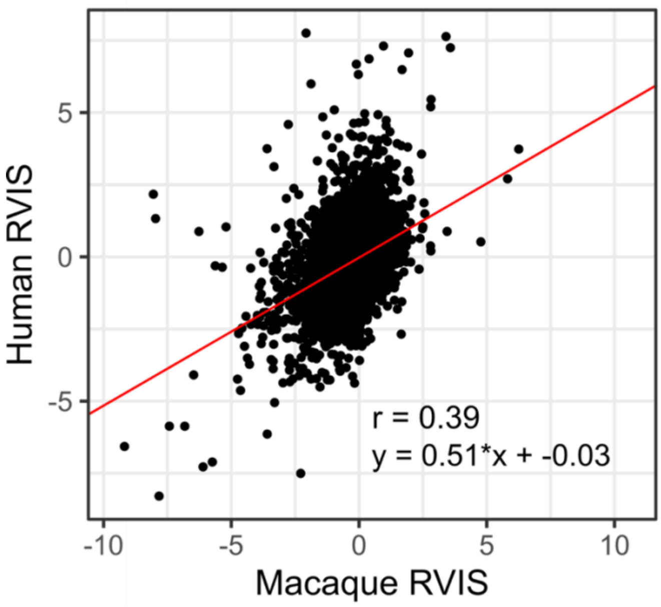

Supplementary Figure 1. Correlation of human and macaque RVIS scores. The correlation was calculated using the macaque and human data (25) across 11898 overlapped genes between macaque and human data after removing the outliers (RVIS > 11). The 95% confidence area ([0.38 - 0.41]) was calculated with a fitted liner regression (red line: Pearson's product-moment correlation = 0.39, *df* = 11,896, $P < 2.2e^{-16}$).

Supplementary Tables

Supplementary Table 1. Summary statistics for all macaque variants before and after QC

Supplementary Table 2: RVIS for different clinical gene set

Supplementary Table 3: Gene over-representation analysis top 2% constrained genes

Supplementary Table 4: GSEA by diagnosis

Supplementary Table 1. Summary statistics for all macaque variants before and after QC

	Pre QC	Post QC
Total variants	40,391,636	37,503,663
Total SNPs	34,741,086	32,507,418
Total Indels	5,650,550	4,996,245
Frameshift mutations	28,102	21,584
Missense mutations	418,065	370,683
Stop gained mutations	12,757	10,035

Supplementary Table 2: RVIS for different clinical gene set

	Mean RVIS (SD), number of genes	Mean RVIS (SD), background no. genes	t	p	β (std. err)	Odds Ratio
Overlapping genes across all four NDDs	-0.52 (0.75) N=101	0 (0.68) N = 18,065	6.93	4.0×10^{-10}	-0.53 (0.07)	0.59
ASD-DISGENET	-0.32 (0.88) N = 793	0.01 (0.67) N = 17,373	10.44	$< 2.2 \times 10^{-16}$	-0.49 (0.04)	0.61
ASD-SFARI	-0.44 (1.10) N = 729	0.02 (0.66) N = 17,437	11.13	$< 2.2 \times 10^{-16}$	-0.63 (0.04)	0.53
Epilepsy	-0.33 (0.79) N = 872	0.01 (0.68) N = 17,294	12.53	$<2.2 \times 10^{-16}$	-0.51 (0.04)	0.60
Schizophrenia	-0.25 (0.85) N = 2,021	0.03 (0.66) N = 16,145	14.40	$<2.2 \times 10^{-16}<$	-0.50 (0.03)	0.61
ID	-0.30 (0.84) N = 1,573	0.03 (0.66) N = 16,593	14.78	$<2.2 \times 10^{-16}$	-0.54 (0.03)	0.58

Supplementary Table 3: Gene over-representation analysis among top 2% constrained genes (N=369)

	Number of genes	Number overlapping	P-value	FDR
ID	1570	89	1.7×10^{-19}	4.3×10^{-19}
ASD	793	50	9.6×10^{-13}	1.2×10^{-12}
ASD-SFARI	729	65	2.8×10^{-24}	1.4×10^{-23}
Epilepsy	871	51	8.9×10^{-12}	8.9×10^{-12}
Schizophrenia	2018	96	5.9×10^{-16}	9.8×10^{-16}

Supplementary Table 4: GSEA by diagnosis

	Number of genes	P-value	FDR	ES
ID	1570	2.1×10^{-47}	1.0×10^{-46}	0.19
ASD	793	2.7×10^{-25}	2.7×10^{-25}	0.19
ASD-SFARI	729	1.2×10^{-33}	2.0×10^{-33}	0.23
Epilepsy	871	7.0×10^{-32}	8.8×10^{-32}	0.21
Schizophrenia	2018	2.6×10^{-44}	6.5×10^{-44}	0.17
